# Hyperoxia‐induced airflow restriction and Renin‐Angiotensin System expression in a bronchopulmonary dysplasia mouse model

**DOI:** 10.14814/phy2.15895

**Published:** 2024-01-01

**Authors:** Jasmine Dowell, Zachary Bice, Ke Yan, Girija G. Konduri

**Affiliations:** ^1^ Medical College of Wisconsin Milwaukee Wisconsin USA

**Keywords:** airflow restriction, bronchopulmonary dysplasia, hyperoxia, mouse model, pediatric lung disease, Renin‐Angiotensin System

## Abstract

Mechanisms underlying hyperoxia‐induced airflow restriction in the pediatric lung disease Bronchopulmonary dysplasia (BPD) are unclear. We hypothesized a role for Renin‐Angiotensin System (RAS) activity in BPD. RAS is comprised of a pro‐developmental pathway consisting of angiotensin converting enzyme‐2 (ACE2) and angiotensin II receptor type 2 (AT2), and a pro‐fibrotic pathway mediated by angiotensin II receptor type 1 (AT1). We investigated associations between neonatal hyperoxia, airflow restriction, and RAS activity in a BPD mouse model. C57 mouse pups were randomized to normoxic (FiO_2_ = 0.21) or hyperoxic (FiO_2_ = 0.75) conditions for 15 days (P1–P15). At P15, P20, and P30, we measured airflow restriction using plethysmography and ACE2, AT1, and AT2 mRNA and protein expression via polymerase chain reaction and Western Blot. Hyperoxia increased airflow restriction P15 and P20, decreased ACE2 and AT2 mRNA, decreased AT2 protein, and increased AT1 protein expression. ACE2 mRNA and protein remained suppressed at P20. By P30, airflow restriction and RAS expression did not differ between groups. Hyperoxia caused high airflow restriction, increased pulmonary expression of the pro‐fibrotic RAS pathway, and decreased expression of the pro‐developmental in our BPD mouse model. These associated findings may point to a causal role for RAS in hyperoxia‐induced airflow restriction.

## Graphical abstract

Longitudinal changes in Renin‐Angiotensin System expression and markers of airflow restriction following neonatal hyperoxia.

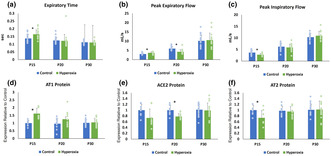

## 1 INTRODUCTION

Neonatal hyperoxia exposure in prematurely born infants can lead to Bronchopulmonary Dysplasia (BPD), a pediatric chronic lung disease (Coalson, 2003, 2006; Li et al., 2018). High airflow restriction is a prominent feature of the abnormal respiratory mechanics that characterize BPD (Clemm et al., 2017; Cockcroft & Davis, 2006; Kim et al., 2006; Northway et al., 1967, 1990; Pelkonen et al., 1997; Royce et al., 2016). Due to their impaired lung function, children with BPD experience higher hospitalization rates, longer hospital stays, and more severe clinical courses when they contract acute illnesses than children without BPD (Baraldi & Filippone, 2007; Greenough et al., 2001; Hong et al., 2016; Rietveld et al., 2004; Shefali‐Patel et al., 2012; Smith et al., 2004). Medical management of BPD is challenging due to limited understanding of the mechanisms by which neonatal hyperoxia leads to high airflow restriction.

The Renin‐Angiotensin System (RAS) is a promising target for investigating the pathophysiology of high airflow restriction in BPD. Key components of RAS are angiotensin converting enzyme (ACE), angiotensin converting enzyme‐2 (ACE2), angiotensin II, angiotensin II type 1 receptor (AT1), and angiotensin II type 2 receptor (AT2). RAS regulates the development and function of many organs and is active in both healthy and diseased lung tissue (Bullock et al., 2001; Gandhi & Uhal, 2016; Johnson et al., 1985; Li et al., 2008; Liu et al., 2009; Ramsay et al., 1997). Its role in flow restriction has been extensively studied in the cardiovascular system, where it causes resistance to blood flow that leads to hypertension. As a result, multiple RAS‐directed therapies have been developed for cardiovascular disease (Li et al., 2014; Schmieder et al., 2007; Te Riet et al., 2015). Growing evidence of RAS activity in the lungs and the availability of RAS‐directed therapies make RAS an attractive therapeutic target for pulmonary disease. Although genetic and developmental RAS modifications have been linked to BPD development and severity (Castro et al., 2014; Kazzi & Quasney, 2005; Ryckman et al., 2012), the mechanistic role of RAS in airflow restriction diseases like BPD has yet to be established.

**FIGURE 1:**
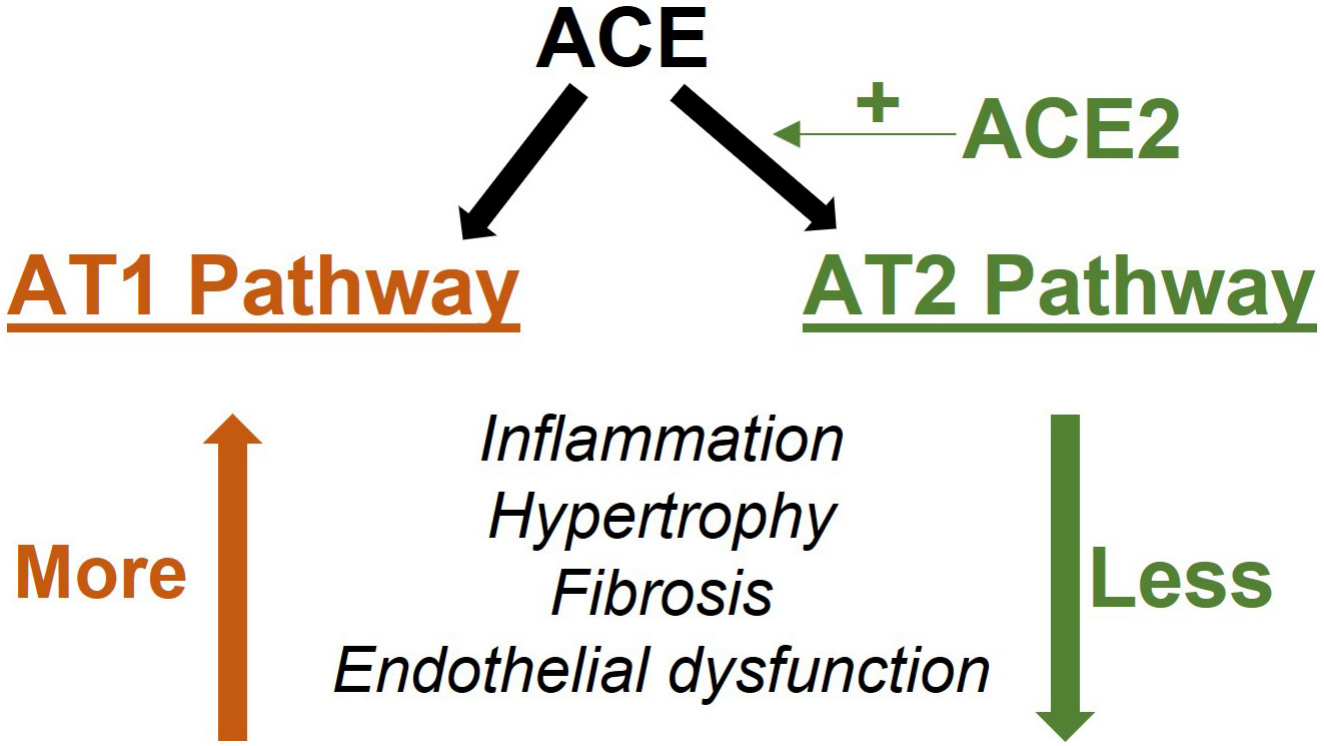
Renin‐Angiotensin System effects.

There are two proposed pathways by which RAS may impact lung development and function. These pathways are mediated by AT1 and AT2 (Figure 1). Prior studies suggest hyperoxia may promote activation of the deleterious AT1 pathway and downregulate activity of the pro‐developmental AT2 pathway (Mohamed et al., 2016; Wagenaar et al., 2013, 2014). However, the degree to which these changes impact airflow restriction is unclear. To address this knowledge gap, our lab employed a hyperoxia‐exposed mouse model of BPD to investigate the relationship between hyperoxia, RAS, and airflow restriction. BPD models from our lab demonstrate cardiopulmonary pathology consistent with BPD, including alveolar simplification and vascular changes (Teng et al., 2021; Yadav et al., 2020). We hypothesized that hyperoxia modulates mRNA and protein expression of RAS components in our mouse model of BPD. We further hypothesized that this abnormal RAS expression is associated with pathologic airflow restriction.

## 2 MATERIALS AND METHODS

### 2.1 Animal care

C57 mouse pups were randomized to normoxic (FiO_2_ = 0.21, room air) or hyperoxic (FiO_2_ = 0.75) conditions for the first 15 days of life (P1–P15). From P15 onward, all animals were in room air. Oxygen concentration was monitored continuously with an oxygen sensor (Drägerwerk AG). Dams were rotated every 24 h between normoxia and hyperoxia litters to avoid maternal oxygen toxicity and control for nutrition quality between litters. All mice had unlimited access to food and water and were maintained on 12‐h day/night cycle. The described studies were approved by the Medical College of Wisconsin Institutional Animal Care and Use Committee and conformed to the current National Institutes of Health guidelines for care and use of laboratory animals.

### 2.2 Plethysmographic measurement of respiratory mechanics and airflow restriction

Whole body plethysmography via FinePointe software (Buxco Small Animal Whole Body Plethysmograph, Data Sciences International, St. Paul, MN) was used to measure the following respiratory indices and flow parameters in awake unrestrained mice according to our lab's previously published rodent protocol (Mouradian et al., 2019): expiratory flow at 50% tidal volume (EF50), peak expiratory flow (PEF), expiratory time (Te), peak inspiratory flow (PIF) and minute ventilation (MV). Plethysmography was performed in two sets of paired normoxia and hyperoxia litters at P15, P20, and P30. Body weight was recorded prior to each measurement.

### 2.3 Renin‐Angiotensin System mRNA and protein measurement

At each timepoint, 8–16 mice were euthanized following plethysmography for whole lung saline‐perfusion and harvest for biochemical analyses. The following RAS components were evaluated at each timepoint: angiotensin converting enzyme (ACE), angiotensin converting enzyme 2 (ACE2), angiotensin II receptor type 1 (AT1), and angiotensin II receptor type 2 (AT2). Real time polymerase chain reaction evaluated in RNA extracted from whole lung homogenate using the Zymo Research Corporation Direct‐zol™ RNA MicroPrep Kit (Fisher Scientific Cat #NC1000412). The Invitrogen SuperScript VILO cDNA Synthesis Kit (Fisher Scientific Cat #11‐754‐050) was then used to synthesize complementary DNA (cDNA) from extracted total RNA. cDNA was combined with beta‐actin housekeeping and RAS target gene primers (RealTimePrimers Actb Cat #VMPS‐96, ACE Cat #VMPS‐78, and ACE2 Cat #VMPS‐79; Sigma Aldrich AT1 Cat #NM_177322 and AT2 Cat #NM_007429) and PowerUp SYBR Green Master Mix (Fisher Scientific, Cat #A25742). The samples were run in triplicate on ABI 196 ViiA7 (Thermo Fischer Scientific) to quantify relative expression of the target genes based on value differences between the target gene and internal control (beta‐actin) using the comparative threshold cycle (Ct) according to the PowerUp SYBR Green RT‐PCR manual. Run protocol parameters included 50°C, 2 min, 95°C, 10 min, and then 40 cycles at 95°C, 15 s, 60°C, 200 60 s. Delta‐Ct was calculated by subtracting the target gene value at Ct 201 from the beta‐actin Ct. Double‐delta‐Ct (2^−ΔΔCt^) calculation determined mRNA quantity.

RAS component protein expression relative to control (unitless) was measured via Western Blot according to our standard lab protocol (Lai et al., 2019). Whole lung lysates were generated from animals in all groups via Bullet Blender homogenization (Next Advance, Inc., Averill Park, NY) in T‐per lysis buffer (Thermo Fisher, REF78510) and a protease/phosphatase inhibitor cocktail (Millipore, 524,624, 524,625, 539,134). We quantified sample protein concentration via Bradford Assay (Bio‐Rad, Cat#5000006) to ensure equivalent protein loading. The volume for 20 μg of each sample was added 1:1 with 2× sample buffer (Bio‐Rad, Cat#1610737) under reducing conditions containing 2‐Mercaptoethanol (Sigma, M3148) and boiled for 5 min at 99°C. Samples were loaded into polyacrylamide gels (Bio‐Rad, Cat#4561086) and resolved via SDS‐PAGE. Gels were transferred to PVDF membranes (Bio‐Rad, Cat#1704156) on the Trans‐Blot Turbo Transfer System (Bio‐Rad). The membranes were blocked with Everyblot Blocking Buffer (Bio‐Rad, Cat#12010020) and then probed with antibodies for the target RAS proteins and beta‐actin (Proteintech ACE Cat #24743‐1‐AP, ACE2 Cat #21115‐1‐AP, AGTR1 Cat #25343‐1‐AP, AGTR2 Cat #BS‐2133, Beta Actin Cat #66009‐1‐Ig) 1:1000 at 4°C overnight. Membranes were subsequently incubated with goat anti‐mouse or goat anti‐rabbit secondary antibodies (Bio‐Rad, Cat#1706515 #1706516) 1:5000 for 1 hour at room temperature. Finally, blots received 5 mL Clarity ECL substrate (Bio‐Rad, Cat # 1705061) and were imaged on the Chemidoc MP Imaging System (Bio‐Rad). Analysis of band optical density processed by Image Lab (Bio‐Rad) to quantify protein expression of the target RAS components, with beta‐actin signal used as the loading control. Antibodies were validated by the presence of a single band at the expected molecular weight.

### 2.4 Statistical analysis

Data is presented as mean ± standard deviation. Normality was determined using Shapiro–Wilk Test. Student's t‐test was used to determine statistically significant differences in plethysmography parameters and RAS mRNA and protein expression between groups at each timepoint with a two‐sided *p*‐value <0.05 used to define significance. Fisher's Exact Test analyzed mortality differences between normoxia and hyperoxia litters.

## 3 RESULTS

### 3.1 Pulmonary function and airflow restriction

#### 3.1.1 Mouse models

**FIGURE 2:**
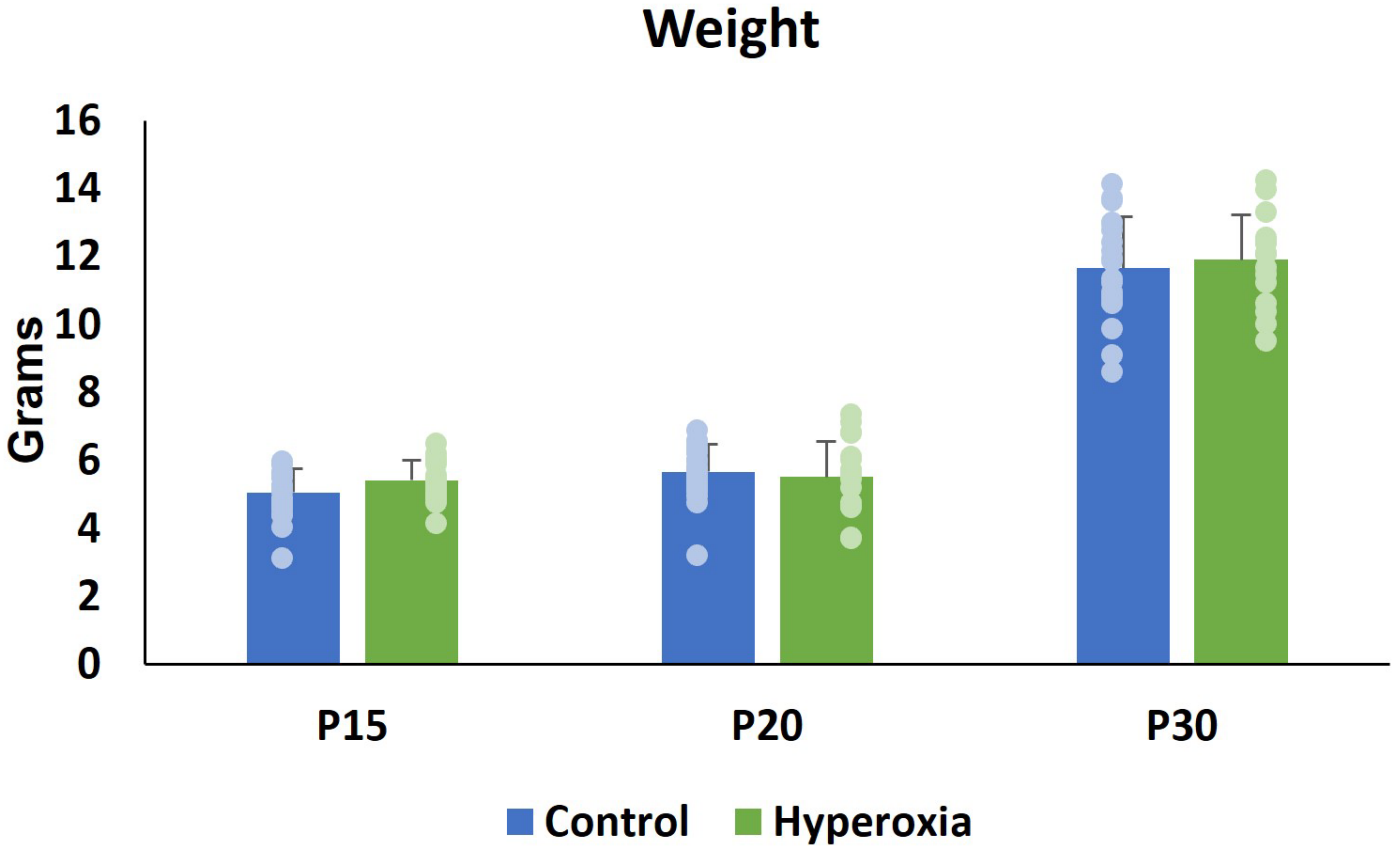
Weight (mean ± SD) in grams at study timepoints. No significant difference in weight between normoxia and hyperoxia animals at P15, P20, P30.

21 mouse pups (14 male, 7 female) were randomized to normoxia and 21 mouse pups to hyperoxia (11 male, 8 female, 2 unknown sex due to death before P15). One pup from normoxia and 5 pups from hyperoxia group died. Mortality rate was not significantly different between two groups (hyperoxia 23.8% vs. normoxia 4.8%, *p* = 0.18). All non‐survivors were male, except the 2 of unknown sex. Weight did not differ significantly between the hyperoxia and normoxia groups at any timepoint (Figure 2). At P30, hyperoxia males weighed more than hyperoxia females. Otherwise, there were no sex‐related differences in growth (Table 1).

**TABLE 1:** Male–female mean weight comparisons.

Group	Age	Weight (g)
Normoxia (Male, Female)	P15	5.1 ± 0.7, 5.0 ± 0.7
	P20	5.7 ± 0.9, 5.7 ± 0.6
	P30	12.0 ± 1.6, 11.0 ± 1.1
Hyperoxia (Male, Female)	P15	5.5 ± 0.7, 5.4 ± 0.5
	P20	5.6 ± 1.2, 5.4 ± 0.8
	P30	12.9 ± 0.9, 10.9 ± 1.0*

*Note*: Weight mean (±SD) did not differ between male and female rats in the Normoxia or Hyperoxia groups throughout the study, except at the P30 timepoint when Hyperoxia males weighed more than Hyperoxia females.

* *p* < 0.05.

#### 3.1.2 Expiratory airflow restriction

**FIGURE 3:**
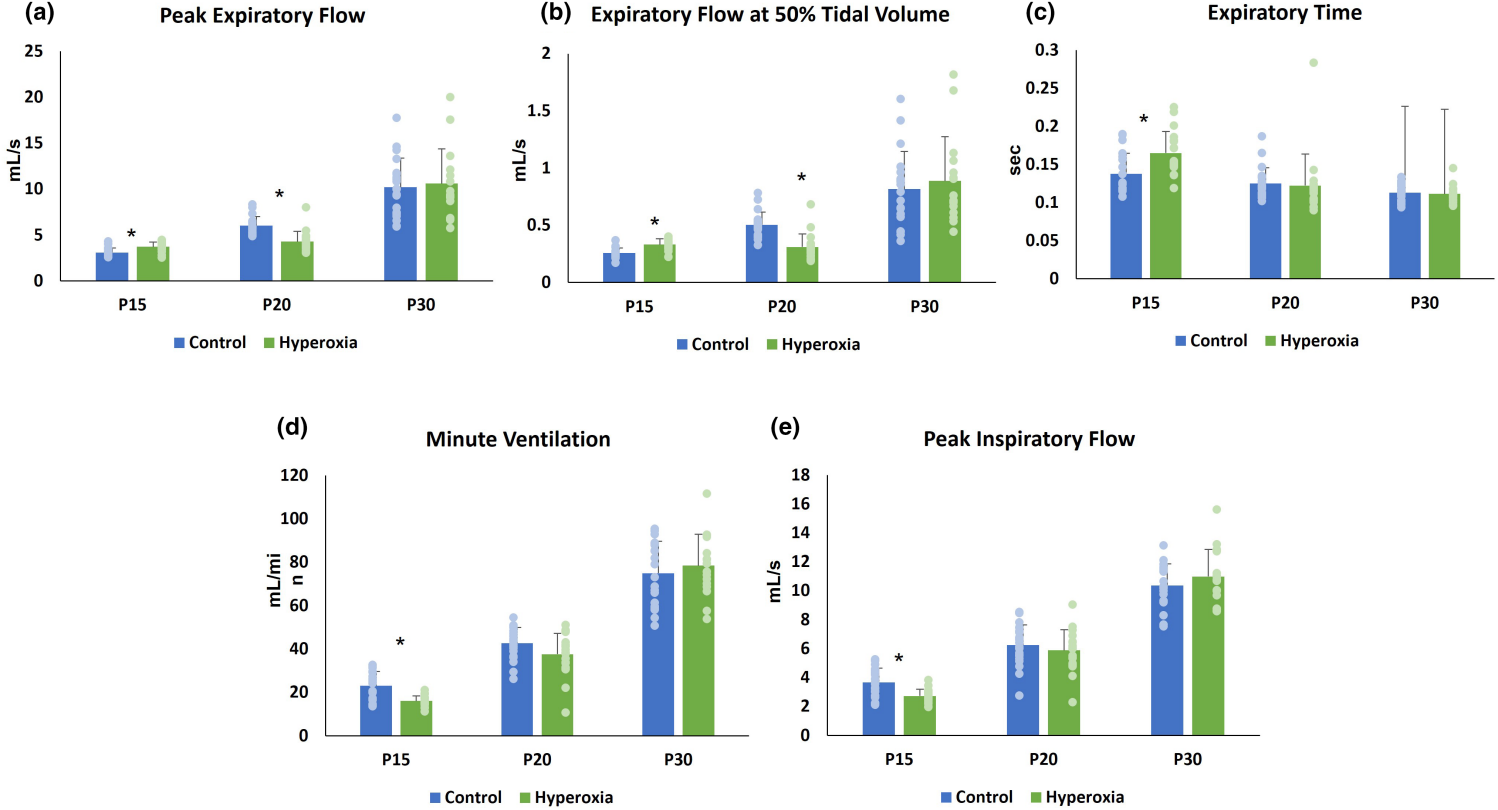
Pulmonary function parameters. Mean ± SD at P15, P20, and P30 for (a) peak expiratory flow, (b) expiratory flow at 50% tidal volume (EF50), (c) expiratory time (Te), (d) minute ventilation (MV) and (e) peak inspiratory flow (PIF). *p < 0.05.

At P15, expiratory flow and time indices were greater in hyperoxia animals, including EF50, PEF, and Te. By P20, hyperoxia animals had significantly lower EF50 and PEF than normoxia animals. Te did not differ at P20. At P30, there was no difference between groups in EF50, PEF, or Te (Figure 3a–c).

#### 3.1.3 Minute ventilation

MV was significantly lower in hyperoxia animals at P15. MV did not differ between groups at P20 (Figure 3d). Minute ventilation is determined by tidal volume (TV) and respiratory rate (RR). TV did not differ between hyperoxia and normoxia animals at any timepoint (*P15* 0.07 ± 0.01 mL vs. 0.07 ± 0.01 mL, *p* = 0.25; *P20* 0.1 ± 0.02 mL vs. 0.11 ± 0.01 mL, *p* = 0.16; *P30* 0.18 ± 0.02 mL vs. 0.17 ± 0.02 mL, *p* = 0.39). RR, measured in breaths per min (bpm), was significantly lower in hyperoxia animals at P15 (228 ± 34 bpm vs. 306 ± 56 bpm; *p* < 0.0001) but did not differ between groups at the later timepoints.

#### 3.1.4 Inspiratory airflow restriction

At P15, hyperoxia animals had lower PIF. PIF did not differ between groups at P20 or P30 (Figure 3e).

*Sex as a Biological Factor and Respiratory Mechanics*: There were no significant male–female differences in inspiratory flow restriction, expiratory flow restriction, or minute ventilation parameters within groups at any timepoint (Table 2).

**TABLE 2:** Male–female pulmonary function comparisons.

Pulmonary function parameter	Age	NORMOXIA (male, female)	HYPEROXIA (male, female)
Minute ventilation (mL/min)	P15	22.8 ± 6.8, 23.3 ± 6.6	16.1 ± 2.8, 15.8 ± 2.0
	P20	43.8 ± 5.7, 40.3 ± 9.8	37.5 ± 12.0, 37.6 ± 5.9
	P30	76.5 ± 15.0, 72.0 ± 14.8	84.5 ± 13.8, 72.4 ± 13.1
Peak expiratory flow (mL/s)	P15	3.1 ± 0.6, 3.0 ± 0.3	3.6 ± 0.6, 3.8 ± 0.4
	P20	6.1 ± 1.2, 5.8 ± 0.6	4.3 ± 0.7, 4.3 ± 1.5
	P30	10.8 ± 3.5, 9.1 ± 2.2	12.2 ± 4.6, 9.0 ± 1.9
Expiratory flow at 50% tidal volume (mL/s)	P15	0.27 ± 0.04, 0.24 ± 0.04	0.32 ± 0.06, 0.34 ± 0.04
	P20	0.53 ± 0.12, 0.46 ± 0.07	0.29 ± 0.09, 0.33 ± 0.15
	P30	0.87 ± 0.36, 0.73 ± 0.25	1.03 ± 0.49, 0.74 ± 0.18
Expiratory time (s)	P15	0.14 ± 0.03, 0.14 ± 0.03	0.17 ± 0.03, 0.16 ± 0.02
	P20	0.12 ± 0.02, 0.13 ± 0.02	0.13 ± 0.05, 0.12 ± 0.01
	P30	0.11 ± 0.01, 0.11 ± 0.01	0.11 ± 0.01, 0.11 ± 0.02
Peak inspiratory flow (mL/s)	P15	3.6 ± 1.0, 3.7 ± 1.0	2.7 ± 0.5, 2.7 ± 0.5
	P20	6.4 ± 1.0, 6.4 ± 1.4	5.9 ± 1.5, 5.9 ± 1.4
	P30	10.6 ± 1.5, 9.8 ± 1.5	11.7 ± 2.1, 10.3 ± 1.4

*Note*: There were no sex‐based differences in pulmonary function parameter means (±SD) among Normoxia and Hyperoxia‐exposed rats at any timepoint.

* *p* < 0.05.

### 3.2 Renin‐Angiotensin System expression

#### 3.2.1 Mouse models

Biochemical analyses were performed on lung tissue from 39 of the mouse pups randomized to normoxia [*P15* 8 pups (2 male); *P20* 11 pups (7 male); *P30* 20 pups (13 male)] and 37 of the mouse pups randomized to hyperoxia [*P15* 9 pups (6 male); *P20* 12 pups (6 male); *P30* 16 pups (8 male)].

#### 3.2.2 RAS mRNA and protein expression (Figures 4, 5, 6)

**FIGURE 4:**
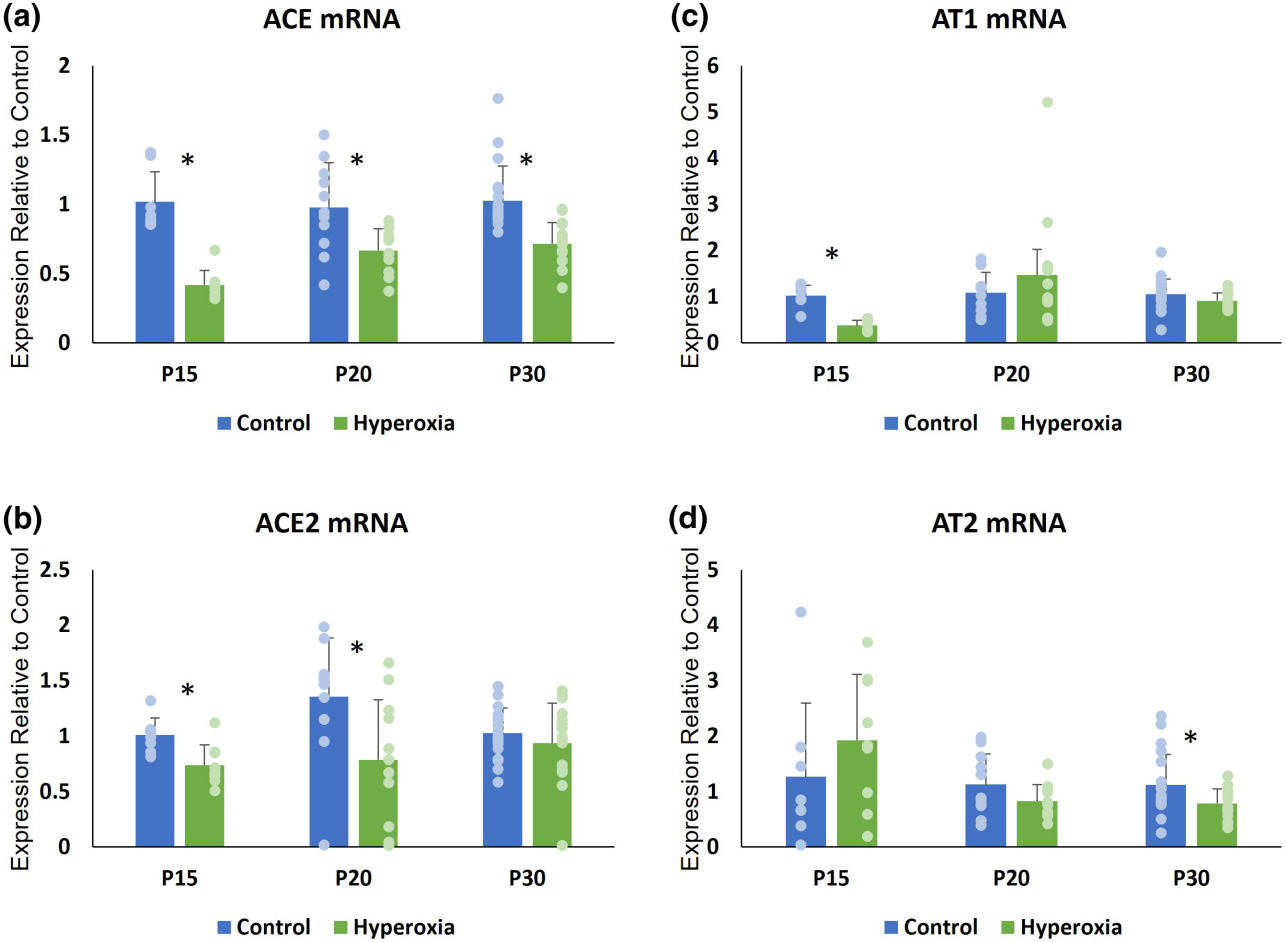
Renin‐Angiotensin System (RAS) mRNA expression. Mean (±SD) mRNA expression at P15, P20, and P30 of (a) angiotensin converting enzyme (ACE), (b) angiotensin converting enzyme‐2 (ACE2), (c) angiotensin‐2 type 1 receptor (AT1), and (d) angiotensin‐2 type 2 receptor (AT2). *p < 0.05.

**FIGURE 5:**
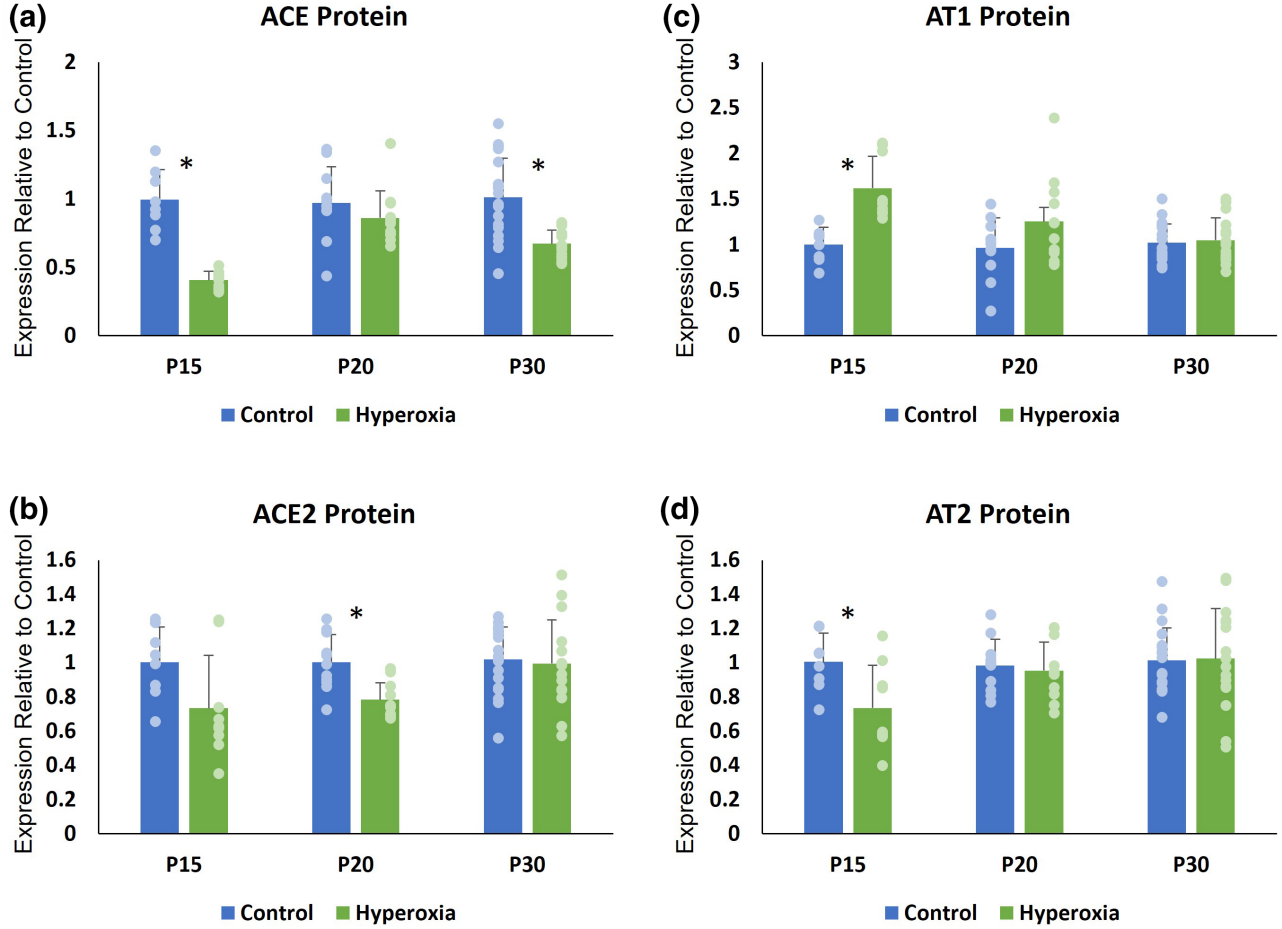
Renin‐Angiotensin System (RAS) Protein Expression. Mean (±SD) protein expression at P15, P20, and P30 of (a) angiotensin converting enzyme (ACE), (b) angiotensin converting enzyme‐2 (ACE2), (c) angiotensin‐2 type 1 receptor (AT1), and (d) angiotensin‐2 type 2 receptor (AT2). *p < 0.05.

**FIGURE 6:**
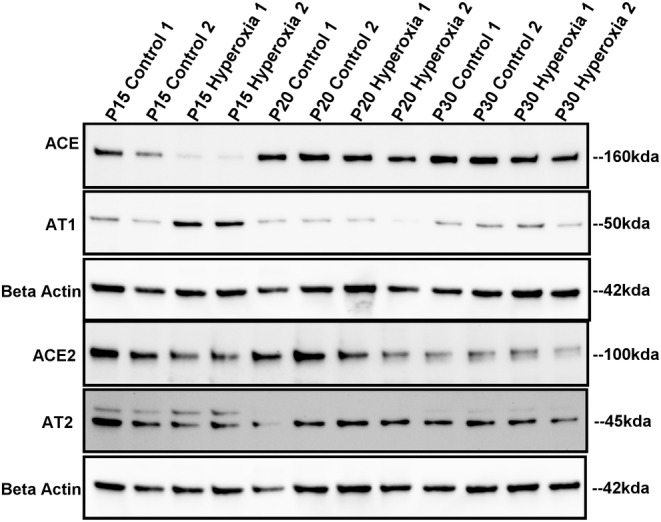
Renin‐Angiotensin System (RAS) Western Blots. Representative longitudinal Western Blots with beta‐Actin control.

At P15, relative mRNA expression was significantly lower in hyperoxia animals for ACE, ACE2, and AT1 while AT2 levels did not differ between groups. Hyperoxia animals had lower relative protein expression of ACE and AT2. ACE2 protein levels also demonstrated a non‐significant trend toward being lower in hyperoxia animals. In contrast, AT1 protein expression was significantly higher in the hyperoxia group.

At P20, hyperoxia animals continued to demonstrate significantly less mRNA expression for ACE and ACE2, but AT1 and AT2 expression did not differ between groups. There was no difference between groups in protein expression of ACE, AT1, or AT2 at P20. However, the hyperoxia group continued to express less ACE2.

At P30, the hyperoxia group expressed less ACE mRNA and ACE protein. There was no difference between groups in mRNA or protein expression of ACE2 and AT1. The hyperoxia group expressed less AT2 mRNA at P30; however, AT2 protein expression did not differ from the normoxia group.

Representative Western Blots are shown in Figure 6.

#### 3.2.3 Sex as a biological variable and RAS expression

Table 3 details comparisons between RAS mRNA and protein expression by sex within the two groups. Sex‐based differences in expression included less AT1 mRNA expression at P15 in normoxia males than normoxia females and more AT2 protein expression at P20 in hyperoxia males than hyperoxia females. At P30, ACE mRNA expression was higher in hyperoxia males than hyperoxia females, though ACE protein expression trended lower in hyperoxia males than hyperoxia females.

**TABLE 3:** Male–Female Renin‐Angiotensin System (RAS) mRNA and Protein Expression Means (±SD).

RAS expression	Age	NORMOXIA (male, female)	HYPEROXIA (male, female)
ACE mRNA	P15	0.90 ± 0.04, 1.06 ± 0.24	0.44 ± 0.12, 0.37 ± 0.05
	P20	1.05 ± 0.31, 0.86 ± 0.36	0.65 ± 0.16, 0.69 ± 0.16
	P30	1.02 ± 0.29, 1.04 ± 0.17	0.79 ± 0.12, 0.64 ± 0.15*
ACE2 mRNA	P15	0.84 ± 0.03, 1.07 ± 0.13	0.80 ± 0.19, 0.62 ± 0.10
	P20	1.44 ± 0.33, 1.22 ± 0.82	0.66 ± 0.61, 0.91 ± 0.49
	P30	0.98 ± 0.22, 1.10 ± 0.23	1.04 ± 0.29, 0.83 ± 0.41
AT1 mRNA	P15	0.76 ± 0.26, 1.11 ± 0.14*	0.41 ± 0.12, 0.32 ± 0.08
	P20	1.05 ± 0.43, 1.17 ± 0.56	1.92 ± 1.76, 1.02 ± 0.50
	P30	0.97 ± 0.25, 1.21 ± 0.42	0.97 ± 0.20, 0.86 ± 0.11
AT2 mRNA	P15	0.92 ± 1.24, 1.38 ± 1.45	1.93 ± 1.35, 1.93 ± 1.03
	P20	1.26 ± 0.56, 0.91 ± 0.55	0.72 ± 0.23, 0.94 ± 0.34
	P30	0.96 ± 0.50, 1.42 ± 0.54	0.78 ± 0.31, 0.79 ± 0.23
ACE Protein	P15	0.91 ± 0.03, 1.02 ± 0.25	0.41 ± 0.05, 0.40 ± 0.10
	P20	1.00 ± 0.31, 0.93 ± 0.19	0.79 ± 0.11, 0.93 ± 0.25
	P30	0.93 ± 0.20, 1.18 ± 0.36	0.62 ± 0.08, 0.72 ± 0.11
ACE2 Protein	P15	0.76 ± 0.15, 1.08 ± 0.16	0.71 ± 0.29, 0.78 ± 0.41
	P20	0.93 ± 0.15, 1.12 ± 0.12	0.77 ± 0.07, 0.79 ± 0.13
	P30	1.04 ± 0.18, 0.98 ± 0.22	1.05 ± 0.32, 0.94 ± 0.17
AT1 Protein	P15	1.11 ± 0.03, 0.97 ± 0.21	1.59 ± 0.37, 1.69 ± 0.37
	P20	0.98 ± 0.38, 0.94 ± 0.26	1.20 ± 0.27, 1.31 ± 0.63
	P30	0.96 ± 0.19, 1.13 ± 0.21	1.07 ± 0.22, 1.03 ± 0.28
AT2 Protein	P15	1.06 ± 0.003, 0.99 ± 0.20	0.67 ± 0.22, 0.87 ± 0.29
	P20	1.00 ± 0.16, 0.95 ± 0.17	1.06 ± 0.15, 0.85 ± 0.11*
	P30	1.04 ± 0.15, 0.96 ± 0.0.26	1.04 ± 0.36, 1.00 ± 0.24

*Note*: Sex‐based differences in RAS expression in the Normoxia group included decreased AT1 mRNA and ACE2 protein in males compared to females. Hyperoxia males expressed increased ACE mRNA at P30 and AT2 protein at P20 compared to females.

* *p* < 0.05.

## 4 DISCUSSION

Hyperoxia‐induced abnormal RAS expression with associated airflow restriction in our mouse model of BPD. Prolonged expiratory time and decreased peak inspiratory flow at P15 were early evidence of elevated biphasic airflow restriction in our model. We also found disordered control of breathing in the hyperoxia animals at P15, evidenced by decreased minute ventilation driven by suppressed respiratory rate. These findings replicate the high airflow restriction and disordered breathing observed in human BPD (Bates et al., 2013; Mammel & Kemp, 2021; Ortiz et al., 2017), which reinforces the validity of our model. The high airflow restriction and abnormal ventilation in our model at P15 was associated with upregulation of the AT1 pathway and suppressed expression of the AT2 pathway components ACE2 and AT2. Subsequently, expiratory flow became restricted, indicated by significantly impaired PEF and EF50 at P20. This worsening airway disease occurred in the setting of persistently decreased ACE2 protein levels. At P30, both RAS expression and airflow restriction returned to control levels as the mice recovered in room air from their hyperoxic insult.

A growing body of research describes a link between RAS and pulmonary pathophysiology and our findings are further evidence of this connection. RAS has previously been implicated in other lung diseases, including idiopathic pulmonary fibrosis, acute respiratory distress syndrome, lung cancer, and COVID‐19 (Hoffmann et al., 2020; Jerng et al., 2007; Marshall, 2003; Sarzani et al., 2020; Tan et al., 2018). Given its known effects on the lungs and the findings in our mouse model, it is plausible that RAS plays a role BPD pathology, as well. In our model, hyperoxia increased expression of AT1 pathway components within RAS, which is associated with inflammation, pulmonary fibrosis, collagen deposition and bronchoconstriction. All of these RAS‐modulated changes could lead to increased airflow restriction similar to that which we observe in BPD. Conversely, downstream effects of the AT2 pathway include decreased fibrosis, healthy lung development through lung branching, and improved pulmonary function. Thus, our finding of increased airflow restriction when AT1 expression was abnormally high and AT2 expression was abnormally low aligns with current knowledge of RAS effects. Notably, our results showed differences in mRNA and protein expression at P15 for RAS components AT1 and AT2. AT1 protein was elevated while mRNA expression was decreased, while the opposite pattern was noted for AT2. Normalized mRNA and protein expression of these components by P20 suggests the downregulation of AT1 mRNA and upregulation of AT2 mRNA at P15 suggests a feedback loop through which the RAS expression corrects as the animals and their lungs recover from hyperoxia.

Our study is novel in its tracking of the post‐hyperoxia trajectories of RAS expression and airflow restriction over time. While other studies have identified that hyperoxia downregulates ACE2 in lung fibroblasts (Oarhe et al., 2015), our study offers a comprehensive evaluation of pulmonary RAS expression in the context of the whole animal and in relation to pulmonary function at multiple timepoints. Importantly, the time course design of our study revealed that hyperoxia‐induced disruption of RAS expression occurs before its effects on expiratory flows. This sequence of altered protein expression preceding functional changes indicates a possible cause‐and‐effect relationship. Additionally, the parallel normalization of RAS expression and airflow restriction we observed as the mice recovered from hyperoxia further points to a potential causal connection between RAS activity and airflow restriction.

Hyperoxia‐induced RAS activity could alter airflow restriction via multiple mechanisms. RAS modulates both bronchial branching and deposition of fibrin and collagen in the bronchial wall (Castro et al., 2014; Gandhi & Uhal, 2016; Uhal et al., 1998). Thus, abnormal RAS activity could result in a simplified bronchial tree structure and thick, narrowed bronchial walls that negatively impact airflow restriction. As RAS normalized during recovery from hyperoxia in our model, the AT2 pathway became more active. AT2 activity may improve airflow restriction by decreasing fibrosis, increasing airway number, and enlarging airway caliber. Whether neonatal hyperoxia alters the airway branching and structure in our model requires further study, including pulmonary imaging and histology of lung tissue to investigate the relationship between hyperoxia, RAS, pulmonary anatomy, and airflow restriction.

Hyperoxia may also affect RAS activity indirectly by modifying the gonadotropic axis. Testosterone is a direct up‐regulator of the AT1 pathway and androgenic modulation of RAS expression has been well‐described (Chen et al., 1992; Ellison et al., 1989; Hilliard et al., 2013; Katz & Roper, 1977; White et al., 2019). Although our mouse model did not demonstrate sex‐based differences in airflow restriction, RAS expression differed somewhat. This suggests a relationship between RAS and airflow restriction may not be directly proportionate and more work is required to determine the magnitude of RAS disturbance required for airway effects. We did find significantly higher mortality in male pups following hyperoxia exposure and that the lung‐protective AT2 pathway was more suppressed in male pups at P20 than in female pups. This finding aligns with clinical observations that male infants suffer a more severe BPD phenotype than female infants and are more than twice as likely to die from the disease (Henderson‐Smart et al., 2006; Naeye et al., 1971; O'Driscoll et al., 2018; Silveyra et al., 2021). Sex differences in severity of BPD pathology have been previously described in hyperoxia‐exposed mouse models (Balaji et al., 2018; Leary et al., 2019; Nguyen et al., 2019); however, more research is required to investigate the role of RAS expression in sex‐based differences in BPD.

Interestingly, ACE was the only RAS component that did not demonstrate normal mRNA expression in hyperoxia animals at P30. ACE is a non‐specific initiator of both RAS pathways. Thus, persistent abnormalities in ACE expression may be a marker of prior insult to the system. ACE downregulation at P30 may also be related to feedback inhibition following the period of upregulation required to achieve overall RAS normalization. Whether ACE expression remains persistently altered at later timepoints requires further studies in older mice following neonatal hyperoxia.

A limitation of our study is death of the sickest hyperoxia animals prior to experiment completion may have contributed to normalization of airflow restriction and RAS expression. Data from these animals could not be accounted for at the P30 timepoint. However, normalization of expiratory time at P20 occurred prior to the death of these study animals and may represent an early marker of recovery in the hyperoxia group. A second limitation is our study design assessed RAS whole lung RAS expression. These findings formed the basis for airway‐specific RAS expression studies currently in progress in our lab.

**FIGURE 7:**
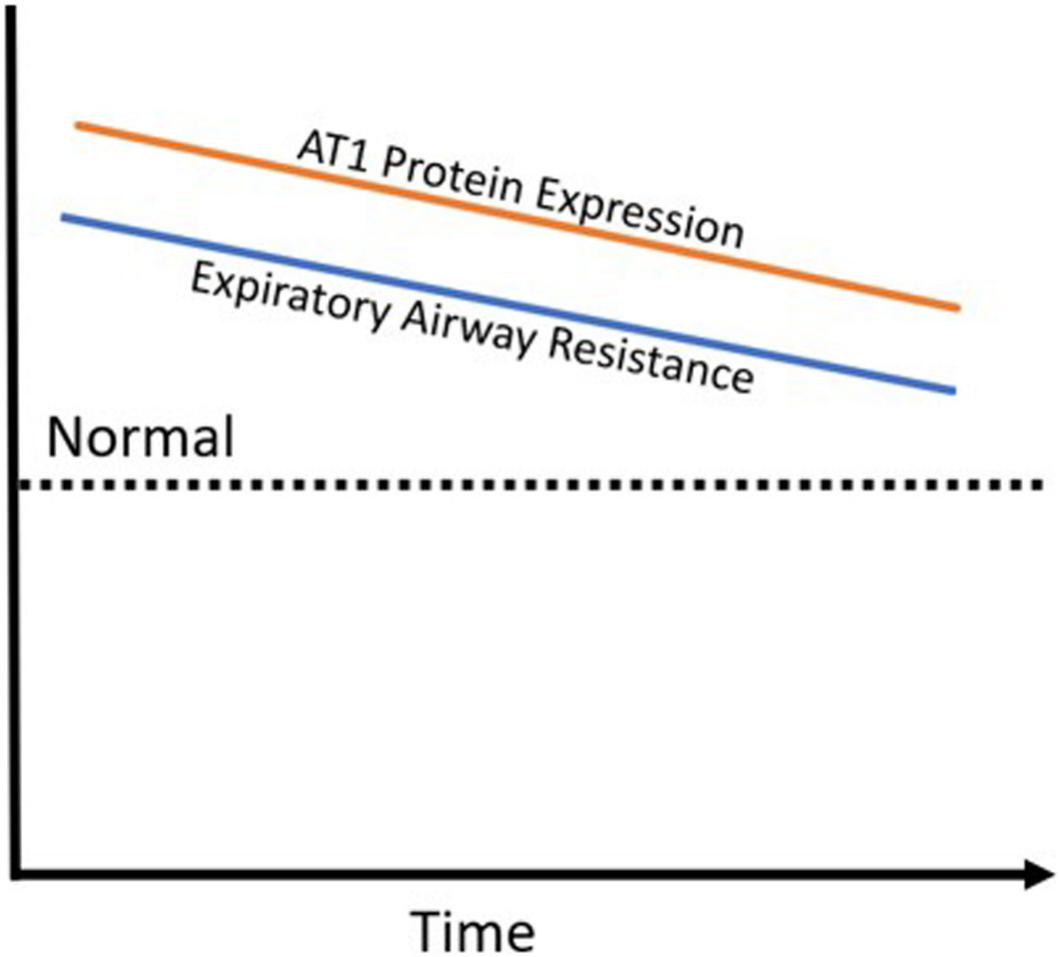
Theoretical model of Renin‐Angiotensin System and airflow restriction trajectories following hyperoxia.

Our study design required lung harvest for RAS analysis at each timepoint and is, therefore, limited by an inability to analyze RAS and airflow restriction data from each animal at every timepoint. However, we were able to use our aggregate data to create a theoretical model of the relationship between changes in RAS profile and airflow restriction over time (Figure 7). The model was created by mapping RAS protein expression at each timepoint alongside expiratory flow at the subsequent timepoint, as we expect airflow restriction to be a downstream effect of dynamic RAS expression. The resultant model suggests airflow restriction trajectory varies directly with AT1 protein. Our future experimental designs will be powered for correlation analysis to allow for refinement and validation of our theoretical model. Confirmation of the model would identify AT1 as a biomarker of airflow restriction severity and recovery following neonatal hyperoxia.

## 5 CONCLUSION

Taken together, our findings indicated that hyperoxia imbalanced the pro‐developmental and pro‐fibrotic RAS pathways in the lungs. This imbalance was associated with development of pathologic airflow restriction that improved as RAS expression normalized, suggesting a causal role for RAS in airway pathology following hyperoxia exposure. These important findings will inform future mechanistic studies of hyperoxia‐induced airway disease. Understanding the mechanisms by which hyperoxia leads to high airflow restriction in BPD is critical for advancing care of affected patients. Our study has identified RAS modulation as a promising target for future research relevant to the clinical sphere, such as whether RAS‐associated airflow restriction is responsive to bronchodilators and RAS‐directed therapies, and investigations into the influence of RAS on pulmonary function in the setting of acute viral illness in BPD.

## AUTHORS' CONTRIBUTIONS

Jasmine Dowell was the primary investigator responsible for study design and execution, data collection/analysis/interpretation and was the major contributor to writing the manuscript. Zachary Bice assisted with animal husbandry, harvesting samples, plethysmography, and biochemical analyses. Ke Yan is the biostatistician responsible for statistical analyses. Girija G. Konduri mentored Jasmine Dowell throughout the research and provided laboratory space and materials. All authors read and approved the final manuscript.

## FUNDING INFORMATION

This research and data analysis was funded by an Innovative Pilot Award from Children's Wisconsin Research Institute (Grant ID 2212597) and Medical College of Wisconsin Department of Pediatrics Internal Funding Award (33013‐156‐030‐6640093) to JD and from GGK funding via 1R01 HL136597 and in part by Advancing a Healthier Wisconsin Endowment Program support.

## CONFLICT OF INTEREST STATEMENT

No financial or non‐financial competing interests to disclose

## ETHICS STATEMENT

This study was approved by the Medical College of Wisconsin Institutional Animal Care and Use Committee (AUA00002268) and conformed to the current National Institutes of Health guidelines for care and use of laboratory animals.

## CLINICAL TRIAL APPROVAL, PERMISSION TO REPRODUCE MATERIAL, AND PATIENT CONSENT

Not applicable.

## Data Availability

The datasets used and/or analyzed during the current study are available from the corresponding author on reasonable request.
